# Intracranial Treatment in Melanoma Patients with Brain Metastasis Is Associated with Improved Survival in the Era of Immunotherapy and Anti-BRAF Therapy

**DOI:** 10.3390/cancers13174493

**Published:** 2021-09-06

**Authors:** Céline Dalmasso, Cécile Pagès, Léonor Chaltiel, Vincent Sibaud, Elisabeth Moyal, Ciprian Chira, Jean Christophe Sol, Igor Latorzeff, Nicolas Meyer, Anouchka Modesto

**Affiliations:** 1 Radiation Oncology Department, Institut Claudius Regaud, Institut Universitaire du Cancer de Toulouse, CEDEX 9, 31059 Toulouse, France; dalmasso.celine@iuct-oncopole.fr (C.D.); moyal.elizabeth@iuct-oncopole.fr (E.M.); chira.ciprian@iuct-oncopole.fr (C.C.); 2 Dermato-Oncology Department, Institut Universitaire du Cancer, CEDEX 9, 31059 Toulouse, France; pages.cecile@iuct-oncopole.fr (C.P.); sibaud.vincent@iuct-oncopole.fr (V.S.); meyer.n@chu-toulouse.fr (N.M.); 3 Biostatistics Department, Institut Claudius Regaud, Institut Universitaire du Cancer de Toulouse, CEDEX 9, 31059 Toulouse, France; chaltiel.leonor@iuct-oncopole.fr; 4 Gamma Knife Unit, CHU–Toulouse-Purpan, 31000 Toulouse, France; sol.jc@chu-toulouse.fr (J.C.S.); ilatorzeff@clinique-pasteur.com (I.L.); 5 Neuro-Surgery Department, CHU de Toulouse–Purpan, 31000 Toulouse, France; 6 Radiation Oncology Department, Oncorad, Clinique Pasteur, 31000 Toulouse, France; 7 Dermatology Department, CHU de Toulouse, Hôpital Larrey, CEDEX 9, 31059 Toulouse, France

**Keywords:** brain metastases, immunotherapy, melanoma, anti-PD-1, anti-BRAF therapy, stereotactic radiation therapy, intracranial treatment

## Abstract

**Simple Summary:**

Melanoma is one of the top three causes of brain metastases, which is still a poor prognostic factor of overall survival. Novel systemic therapies have changed the prognosis of patients and the place of local intracranial treatment, surgery and/or radiotherapy in this era remains unclear. We evaluated the incidence of brain metastasis in melanoma patients in a large retrospective French cohort who received immunotherapy and/or targeted therapy for and identified prognostic factors. Local intracranial treatment is statistically significantly associated with improved overall survival through comparable groups in terms of age, number of BM, BRAF status and systemic treatment. The question of administering local treatment, even for more than one metastasis, such as stereotactic radiotherapy, should be addressed at the diagnosis of brain metastasis while introducing systemic treatment such as immunotherapy. Three prospective trials evaluating additional SRT in combination to ipilimumab and nivolumab association are ongoing.

**Abstract:**

Metastatic melanoma patients are at high risk of brain metastases (BM). Although intracranial control is a prognostic factor for survival, impact of local (intracranial) treatment (LT), surgery and/or radiotherapy (stereotactic or whole brain) in the era of novel therapies remains unknown. We evaluated BM incidence in melanoma patients receiving immune checkpoint inhibitors (ICI) or anti-BRAF therapy and identified prognostic factors for overall survival (OS). Clinical data and treatment patterns were retrospectively collected from all patients treated for newly diagnosed locally advanced or metastatic melanoma between May 2014 and December 2017 with available BRAF mutation status and receiving systemic therapy. Prognostic factors for OS were analyzed with univariable and multivariable survival analyses. BMs occurred in 106 of 250 eligible patients (42.4%), 64 of whom received LT. Median OS in patients with BM was 7.8 months (95% CI [5.4–10.4]). In multivariable analyses, LT was significantly correlated with improved OS (HR 0.21, *p* < 0.01). Median OS was 17.3 months (95% CI [8.3–22.3]) versus 3.6 months (95% CI [1.4–4.8]) in patients with or without LT. LT correlates with improved OS in melanoma patients with BM in the era of ICI and anti-BRAF therapy. The use of LT should be addressed at diagnosis of BM while introducing systemic treatment.

## 1. Introduction

Immune checkpoint inhibitors (ICI) and BRAF + MEK inhibitors combinations have dramatically changed the management of metastatic melanoma patients and improved outcomes [1,2]. However, melanoma is still the third most common cancer type associated with brain metastasis (BM), after lung and breast carcinomas, even in cases of complete extracranial disease control [3]. It has been estimated that a proportion of 20% present brain metastasis at baseline and 50% of patients display intra-cranial progression in the course of metastatic disease (up to 80% of patients in autopsy series) [4,5].

To date, several phases II studies of targeted therapy or immunotherapy have been reported in melanoma patients with central nervous system metastases. [3,6,7,8,9].

In the case of asymptomatic BMs, ipilimumab plus nivolumab is considered as the standard treatment leading to high overall response rate (ORR) in the brain (51–54%) [6,9]. Although dabrafenib plus trametinib shows clear activity in patients with BRAF V600-mutated asymptomatic BMs (ORR 58%), the durability of these responses appears shorter, with a 1-year PFS of 19% [3]. Therefore, there is consensus to use combination immunotherapy in patients with BMs irrespective of BRAF mutational status [10].

Despite recent advances in the management of systemic disease, the occurrence of BMs remains associated with a significantly worse prognosis [11,12,13,14]. BM melanoma patients are a heterogeneous subgroup with a variable prognosis. Prognosis is associated with risk factors including ECOG ≥ 1, presence > 3 BM, extracranial tumour load and age >70 years [13,15,16]. Given the small size of the lesions in both BM trials, the impact of local treatment in the era of novel therapies remains unclear [17,18,19]. We conducted a retrospective study to assess potential prognostic factors, especially outcomes according to administration of local (intracranial) treatment (LT) with surgery and/or radiotherapy for BM in melanoma patients receiving ICI or anti-BRAF therapy.

## 2. Materials and Methods

### 2.1. Study Design and Data Collection

As part of an institutional board-approved study, all consecutive patients treated at the Institut Universitaire du Cancer and CHU de Toulouse, France between May 2014 and December 2017 for newly diagnosed, locally advanced or metastatic melanoma with available tumour status for BRAF mutation were identified by the treatments’ database which excluded patients who did not received systemic treatment. Data on demographics, clinical pathology and molecular analyses, including prognostic factors according to the updated melanoma-molGPA score [15,20] were retrospectively extracted from prospectively collected medical records.

#### Treatments

For each patient, therapeutic strategy was discussed by the institutional melanoma tumour board. Choice of systemic therapy was based on tumour BRAF status and previously received systemic therapies. Regimens administered included a BRAF + MEK inhibitor (BRAFi + MEKi) combination consisting of dabrafenib 150 mg twice daily plus trametinib 2 mg daily. Patients receiving the BRAFi + MEKi regimen with concurrent radiotherapy were instructed to interrupt treatment for three days before and during radiotherapy. Drug intake was resumed the day after. Anti-PD-1 therapy consisted of nivolumab (3 mg/kg, every 2 weeks) or pembrolizumab (2 mg/kg, every 3 weeks). The anti-CTLA-4 therapy ipilimumab was administered as four infusions of 3 mg/kg, every 3 weeks. Both anti-PD-1 and anti-CTLA-4 were administered concomitantly with radiation therapy. In the event of progression after ICI or BRAF therapy, chemotherapy consisting of dacarbazine, fotemustine or temozolomide was administered. Patients with BM received either systemic therapy with or without LT, consisting of surgery, radiation therapy (whole brain radiation therapy [WBRT], or definitive or adjuvant stereotactic radiation therapy [SRT]) taking into account the size and number of BM, the medical condition of the patient and the status of the extra-cranial disease. Surgery was planned in cases of voluminous (>3 cm) or symptomatic BM, particularly in case of inaugural BM present at metastatic diagnosis. SRT was selected in cases of limited intracranial disease (≤3 BM) and was performed at a dose of 20 Gy delivered in a single fraction, or 27 Gy delivered in three fractions for BM with a diameter >2 cm (Gamma Knife, Elekta^®^ or Novalis, Varian^®^) prescribed to the 80% isodose. Adjuvant SRT was delivered to the tumour resection’s bed at a dose of 27 Gy or 30 Gy delivered in three or five fractions, respectively (Novalis, Varian^®^). Alternatively, WBRT was administered at a dose of 30 Gy or 37.5 Gy in 10 to 15 fractions in the event of multiple simultaneous BM (≥4). Combined treatment was defined as immunotherapy or BRAFi delivered within the three months preceding or 11 days following LT respectively (corresponding to five half-lives) [18,21]. All patients underwent systematic follow-up including brain CT scan or Gadolinium-enhanced MRI every 3 months after the first metastasis was diagnosed or, in the event of neurological symptoms, during the course of the disease. Toxicity related to LT was reported according to the Common Terminology Criteria for Adverse Events (CTCAE), version 4.3.

### 2.2. Statistical Analysis

Patient characteristics were presented with descriptive statistics. Qualitative variables were summarized using the frequency and percentage for each category. Continuous variables were summarized using median and range. Differences between groups were assessed using Chi-square or Fisher’s exact test for categorical variables and Kruskal–Wallis test for continuous variables. Overall survival (OS) was defined as the time from the first BM to death or last follow-up (censored data) and was estimated with the Kaplan-Meier method and presented with 95% confidence intervals (CI).

Univariable analyses were performed using the log-rank test. Qualitative ssignificant variables on univariable analyses and clinically relevant variables were included in the multivariable analysis, which was performed using the Cox proportional hazards model and presented with hazard ratios (HR) and the 95% CI. For all statistical tests, differences were considered significant at the 5% level. All statistical analyses were performed using STATA 13.0 software.

### 2.3. Ethics

All of the regulatory procedures required to comply with the laws in force in France were complied with (declaration to the Health Data Hub under MR-004).

## 3. Results

### 3.1. Patient Population

Of the 330 patients screened for this study, 80 were excluded from analyses (27 of whom did not receive systemic therapy and 53 who were lost to follow-up), resulting in 250 eligible patients. With a median follow-up of 28 months (95% CI 25–33), 106 (42.4%) patients presented BMs during the course of their disease. Demographic, disease and treatment characteristics of the 250 patients are presented in Table 1 according to the presence of BM or not. Among the 106 patients with BM, 29.2% presented symptoms at time of BM diagnosis, including intracranial hypertension (*n* = 13), sensitive-motor deficit (*n* = 12) and epilepsy (*n* = 7).

### 3.2. BM and Survival

**Figure 1:**
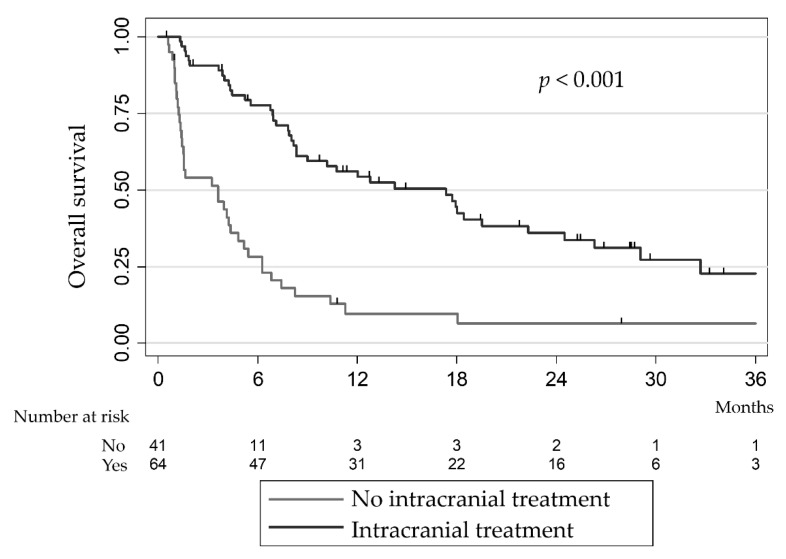
Kaplan-Meier estimate of overall survival according to local intracranial treatment for melanoma brain metastasis in patients with brain metastases.

The 106 patients with BM showed a median OS of 7.8 months (95% CI [5.4–10.4]). In this population, age < 70 years at first BM, LDH levels ≤ 1 ULN (upper limit of normal), three or fewer BM and LT were significantly correlated with favourable prognosis in the univariable analysis (Table 2). Multivariable analysis showed that LT (HR = 0.21, 95% CI [0.10–0.43], *p* < 0.01), ECOG PS ≤ 2 (HR = 0.40% CI [0.18–0.88], *p* = 0.02) and LDH ≤ 1 ULN (HR = 0.44, 95% CI [0.21–0.95], *p* = 0.04) were significantly associated with better OS (Table 3). Among patients who had not received LT, median OS was 3.6 months (95% CI [1.4–4.8]) and the 12-month OS rate was 9.6% (95% CI [2.8–21.7]), compared to 17.3 months (95% CI [8.3–22.3]) and 56.1% (95% CI [42.8–67.5]), respectively, in patients who had undergone LT (Figure 1; *p* < 0.001). As previously reported, the addition of fractioned SRT to anti-PD-1 treatment after initial progression led to a complete extracranial and intracranial response in one patient via an abscopal effect [22].

### 3.3. BM Patients with or without LT

Sixty-four of the 106 patients with BM (60.4%) received LT, with a median of follow-up of 28.5 months (95% CI [25.2;33.2]) versus 27.8 months (95% CI [1 0.7;NR]) for patients who did not receive LT. Local treatment consisted of definitive SRT (*n* = 28, 43.8%), WBRT (*n* = 21, 32.8%) or surgery (*n* = 16, 25%), which was followed by radiation therapy of the operating bed using SRT for eight patients and WBRT for one patient. Concurrent systemic therapy was administered to 44 of the 64 (68.8%) patients, including ipilimumab (*n* = 6, 13.6%), anti-PD-1 (*n* = 19, 43.2%), targeted therapy (*n* = 15, 34.1%), or chemotherapy (*n* = 4, 9.1%). After the diagnosis of BM, 61.9%, 53.6% and 57.1% of patients who respectively underwent surgery, SRT or WBRT received at least one line of ICI during the process of the disease, and 33.3%, 35.7% and 42.9% of patients who respectively underwent surgery, SRT and WBRT received at least one line of targeted therapy during the process of the disease (Table 4).

Seventeen patients underwent LT after a 2 month interval; meanwhile they received systemic treatment.

Patients with BM who underwent LT were more likely to present with neurological symptoms at diagnosis of BM (*p* < 0.01), normal LDH (*p* < 0.01) and be systemic treatment naïve (inaugural BM) than patients who did not (*p* = 0.04) (Table 3). Patients who underwent LT were less likely to receive immunotherapy before BM appearance than pateints who did not have LT (*p* = 0.03). There was no difference in receiving previous iBRAF and/or iMEK for BRAF-mutated patients before BM.

### 3.4. Safety of LT

No grade 4 or 5 radiation or surgical related toxicities were reported in the 64 patients who underwent LT. No events associated with the surgical procedure were reported. Details of radiation-induced side effects are provided on Table 5.

## 4. Discussion

We herein report on outcomes from a large retrospective single-institution cohort of metastatic melanoma patients treated during the era of novel systemic therapies. Of the 250 patients with metastatic melanoma, 42.4% presented BMs during the course of their disease, associated with a poor median OS (7.8 months), which is consistent with previously reported rates [3,7,12,15]. The median OS of 3.6 months (95% CI [1.4–4.8]) and a 12-month OS rate of 9.6% (95% CI [2.8–21.7]) in patients who had not received LT compared to median OS of 17.3 months (95% CI [8.3–22.3]) and a 12-month survival rate of 56.1% (95% CI [42.8–67.5]) in patients who received LT (*p* < 0.001), shows that LT significantly favours survival. In multivariable analyses, LT remained significantly associated with improved OS (HR 0.21, 95% CI [0.1–0.43]; *p* < 0.001). Despite major therapeutic advances, BM remained associated with a poorer prognosis. Moreover, only a few data are available on the treatment of melanoma BM, as these patients were systematically excluded from clinical trials [23]. The rare clinical trials addressing the treatment of melanoma patients with BM did not evaluate combined strategies including LTs. These studies also illustrated the heterogeneity of the melanoma BM subpopulation and the major prognostic differences between BM patients. This makes it difficult to extrapolate results from prospective trials in relation to highly selected patients and strengthens the need of additional local treatment strategies to improve intracranial control even in the era of recent effective systemic treatment. In our study, using prognostic factors from the melanoma-molGPA score, we found comparable survival rates to those previously reported by Sperduto et al., and patients were well balanced according to melanoma molGPA criteria whether they received LT or not [15,20]. So far LT has not been used to define the criteria of the MolGPA score.

Survival of our unselected patients with BM was similar to that reported in the phase II COMBI-MB study (median OS ranged from 11 months for asymptomatic patients without LT to 24 months for asymptomatic ones with previous LT) and in line with the non-reached median OS in BM patients receiving nivolumab plus ipilimumab in two phase II studies [3,6]. Interestingly, in another phase II study, BREAK-MB, evaluating dabrafenib as a single agent therapy for patients with melanoma BM, the subgroup of BM patients who did not receive LT was also associated with lower survival rates than those receiving LT (median of OS 4 vs. 8 months respectively) [7]. Vosoughi et al. also reported in a retrospective analysis that LT (craniotomy or SRT) was independently associated with improved OS (17 months after LT versus 12 months for the entire cohort from time to BM diagnosis) [12]. Together these studies highlight that intracranial control including local treatment is associated with improved overall survival [3,6,12].

The role of local treatment for single BM patients has been investigated across various tumours. Even in cases necessitating initial surgery (i.e., symptomatic lesions or inaugural diagnosis), adjuvant irradiation, either with WBRT or fractioned-SRT, can add benefit to local control [24,25,26]. SRT has shown better results in terms of quality of life whereas WBRT brings cognitive deterioration which is no more compatible with prolonged survival [27,28,29]. In a recent randomized phase III study, WBRT after local treatment for one to three melanoma BMs did not provide any clinical benefit, distant intracranial control, survival or preservation of quality of life, which differs from other histologies, highlighting the need for adapted studies [30]. Median of OS was 16 months in the entire population, which is comparable to our results.

In the latest German guidelines, LT (but not WBRT) is recommended for all BM in case of oligometastatic disease [31]. Definitive or adjuvant SRT delivered in single or multiple fractions, depending on the size of the BM, is recommended in the first management step when technically feasible [19,26,32,33]. In the randomised phase II COMET trial, ablative SRT of oligo-metastatic patients, including those with BM derived from various tumours, was associated with improved OS (13 months) [33].

Many retrospective studies reported enhanced local control when radiotherapy was delivered in combination with immunotherapy, providing further evidence for a synergistic effect of ICIs and radiation therapy [34,35,36].

Several preclinical studies reported that the combination of low-dose fractionated radiation therapy and PD-1/PD-L1 axis inhibitors activates cytotoxic T cells infiltrating and immediately surrounding the tumour [37]. Recent studies reported clinical results of this synergy, leading to an immunotherapy-mediated abscopal effect [9,17,34,35,36]. In a retrospective analysis of patients with BM from various tumours, patients who received immunotherapy in combination with fractionated SRT had significantly better 1-year local control (96% vs. 78%, *p* = 0.02) and improved distant intracranial disease-free survival (62% vs. 51%, *p* = 0.007) than those who did not [35]. In a retrospective studies of 46 patients, better results in OS and local recurrence were reported if immunotherapy was delivered during (or before) SRT than after [37]. In another retrospective series of 25 patients, SRT delivered in less than 30 days after immunotherapy yielded a trend toward a better brain control [38]. Nevertheless, these small series lack the power to be included in the melanoma BM strategy.

For Braf-mutated tumours, the combination of iBraf + iMek in the first line is challenged by the bi-immunotherapy anti CTLA4 + antiPD1/PDL1, especially for an asymptomatic and slow progressive disease. Either with ICI, or with targeted therapies, bitherapy is superior to monotherapy [31]. The ongoing clinical trials SECOMBIT and IMMUNOCOBIVEN will help to define the best therapeutic sequence in Braf-mutated patients [39,40].

We observed very few adverse events when LT was combined with systemic therapy, confirming the previously reported safety of such a therapeutic combination [22,41]. Safety of combination therapy in the literature is heterogeneous; however almost all authors agree that the putative slightly increased risk is manageable, particularly in light of the likely benefit on efficacy and survival [42,43]. In our study, 10% of the patients developed radionecrosis, which was, in most cases, reversible and manageable with transient steroid therapy and consistent with previously reported results [35]. However given this non-negligible risk, and the difficulty of distinguishing it from local failure, multimodal MRI is strongly recommended during follow-up [44]. Interestingly some authors previously reported that fractionated SRT can reduce the incidence of radionecrosis compared to a single fraction, without compromising local benefit [45].

In addition to its retrospective nature, our study presents a number of limitations. Although the two treatment subgroups (LT vs. no LT) were well balanced with respect to age, number of BM, BRAF status and systemic treatment, patients receiving LT presented significantly more symptomatic BM. Interestingly, despite this negative clinical feature, this subgroup had a better prognosis, supporting our hypothesis that LT is associated with improved survival. Furthermore, a large proportion of our patients (32%) receiving LT was treated with WBRT, which has been reported to be associated with worse local control compared with SRT [28,32], which may have lowered the potential survival benefit of LT. In recent years, a growing body of evidence supports adjuvant or definitive SRT as an alternative to WBRT as an initial therapy approach [10].

## 5. Conclusions

We report on a large series of 106 consecutive melanoma patients with BM receiving systemic therapy of ICI or anti-BRAF inhibitors. Intracranial control has been a recognised prognostic factor of the utmost importance in BM patients for some time, including in the current era of immunotherapy and targeted therapy. Our multivariable analysis confirmed that intracranial treatment was significantly associated with improved OS. The combination of radiation therapy and immunotherapy was well tolerated, avoiding delay for systemic treatment initiation. The question of administering LT such as stereotactic radiotherapy should be addressed at diagnosis of BM, while introducing systemic treatment such as immunotherapy. Three prospective trials; NCT03340129, NCT0210775 and NCT03728465 evaluating additional SRT in combination to ipilimumab and nivolumab association are ongoing [46,47].

## Figures and Tables

**Table 1:** Patient characteristics according to the presence of BM, at the diagnosis of 1st metastasis.

	Total *n* = 250	Patients with BM *n* = 106	Patients without BM *n* = 144
Age at 1st metastasis in years			
Median	66	65	66
(range)	(27–90)	(27–89)	(28–90)
Sex			
Male	141 (56.4%)	64 (60.4%)	77 (53.5%)
female	109 (43.6%)	42 (39.6%)	67 (46.5%)
Primary site of metastasis			
Limbs	88 (35.2%)	43 (40.6%)	45 (31.3%)
Trunk	66 (26.4%)	27 (25.5%)	39 (27.1%)
Head or neck	34 (13.6%)	15 (14.2%)	19 (13.2%)
Mucosa	21 (8.4%)	4 (3.8%)	17 (11.8%)
Unknown	31 (12.4%)	15 (14.2%)	16 (11.1%)
Other	10 (4.0%)	2 (1.9%)	8 (5.6%)
Molecular characteristics			
BRAF	105 (42.0%)	52 (49.1%)	53 (36.8%)
NRAS	38 (15.2%)	15 (14.2%)	23 (16.0%)
BRAF + NRAS	2 (0.8%)	0 (0.0%)	2 (1.4%)
cKIT	11 (4.4%)	6 (5.7%)	5 (3.5%)
BRAF + cKIT	1 (0.4%)	1 (0.9%)	0 (0.0%)
Wildtype	88 (35.2%)	31 (29.2%)	57 (36.9%)
LDH			
≤1 ULN	79 (52.7%)	32 (50%)	47 (54.7%)
>1 ULN	71 (47.3%)	32 (50%)	39 (45.3%)
Missing	100	42	58

BM: brain metastasis, LDH: lactate dehydrogenase, ULN: upper limit of normal. *p* value statistically significant, <0.05.

**Table 2:** Univariable and multivariable analysis of OS in patients with BM.

	Univariable			Multivariable		
	12-Month Survival	[95% CI]	*p* Value	HR	[95% CI]	*p* Value
Age at first BM						
<70 years	49.6%	[36.6; 61.3]		1		
≥70 years	20.1%	[9.1; 34.1]	**0.04**	1.07	[0.52–2.22]	0.86
ECOG PS						
2/3/4	25.0%	[9.1; 44.9%]		1		
0/1	41.8%	[30.5; 52.7]	0.21	0.40	[0.18–0.88]	**0.02**
LDH						
>1 ULN	16.2%	[5.9; 30.9]		1		
≤1 ULN	57.8%	[37.6; 73.5]	**<0.01**	0.44	[0.21–0.95]	**0.04**
Intracranial local treatment						
No	9.6%	[2.8; 21.7]		1		
Yes	56.1%	[42.8; 67.5]	**<0.01**	0.21	[0.10–0.43]	**<0.01**
Number of BM						
≤3	49.6%	[36.4; 61.5]		1		
>3	22.8%	[11.2; 36.8]	**<0.01**	1.61	[0.81;3.21]	0.17
Maximal diameter						
≤10 mm	50.8%	[30.6; 67.8]				
>10 mm	41.6%	[27.4; 55.3]	0.35			
BRAF mutation						
Absent	31.2%	[19.1; 44.2]				
present	45.6%	[31.4; 58.8]	0.70			
Gender						
Male	37.0%	[25.2; 48.8]				
Female	41.1%	[25.2; 56.0]	0.94			

BM: brain metastases, CI: confidence interval, HR: Hazard ratio, ECOG PS: Eastern-Cooperative-Oncology-Group Performance Status, LDH: lactate dehydrogenase. Bold police: *p* value statistically significant, <0.05.

**Table 3:** Characteristics in patients with BM, according to intracranial treatment.

	Patients with BM *n* = 106	Patients without Local Treatment *n* = 42	Patients with Local Treatment *n* = 64	*p* Value
Age at first BM in years	66.0	68.5	65.0	0.62
Median (range)	(27.0–90.0)	(27.0–90.0)	(28.0–89.0)	
Gender				
Male	64 (60.4%)	28 (66.7%)	36 (56.3%)	0.28
Female	42 (39.6%)	14 (33.3%)	28 (43.8%)	
Molecular characteristics				
BRAF	53 (50%)	19 (45.2%)	34 (53.1%)	0.43
NRAS	15 (14.2%)	5 (11.9%)	10 (15.6%)	0.59
c-KIT	7 (6.6%)	3 (7.1%)	4 (6.3%)	1.0
Wildtype	31 (29.2%)	16 (38.1%)	15 (23.4%)	0.10
LDH				
≤1	32 (50%)	8 (29.6%)	24 (64.9%)	**<0.01**
>1	32 (50%)	19 (70.4%)	13 (35.1%)	
Missing	42	15	27	
ECOG PS (*n* = 101)				
0/1	80 (79.2%)	31 (77.5%)	49 (80.3%)	0.7320
2/3/4	21 (20.8%)	9 (22.5%)	12 (19.7%)	
missing	5	2	3	
Extracranial disease at first metastasis				
No	5 (4.7%)	0	5 (7.8%)	
Yes	101 (95.3%)	42 (100%)	59 (92.2%)	0.15
Node	61 (57.5%)	26 (61.9%)	35 (54.7%)	0.46
Liver	27 (25.5%)	16 (38.1%)	11 (17.2%)	**0.02**
Lung	51 (48.1%)	20 (47.6)	31 (48.4%)	0.93
Bone	20 (18.9%)	10 (23.8%)	10 (15.6%)	0.29
Inaugural * BM	14 (13.2%)	2 (4.8%)	12 (18.8%)	**0.04**
Metachronous ** BM	92 (86.8%)	40 (95.2%)	52 (81.3%)	
Neurologic symptoms at diagnosis of BM				
Yes	31 (29.2%)	4 (9.5%)	27 (42.2%)	**<0.01**
No	75 (70.8%)	38 (90.5%)	37 (57.8)	
Number of BM				
1	45 (42.9%)	15 (35.7%)	30 (47.6%)	0.45
2–4	25 (23.8%)	12 (28.6%)	13 (20.6%)	
>4	35 (33.3%)	15 (35.7%)	20 (31.7%)	
Missing	1	0	1	
Maximal diameter (mm) Median (range)	13.0 (1.0–60.0)	11 (1.0–43.0)	14.5 (1.0–60.0)	0.08
Previous systemic treatment				
No	49 (46.2%)	12 (28.6%)	37 (57.8%)	**<0.01**
Yes	57 (53.8%)	30 (71.4%)	27 (42.2%)	
Previous immunotherapy				
No	66 (62.3%)	21 (50%)	45 (70.3%)	**0.03**
Yes	40 (37.7%)	21 (50%)	19 (29.7%)	
For BRAF patients: previous iBRAF/iMEK				
No	28 (52.8%)	7 (36.8%)	21 (61.8%)	0.08
Yes	25 (47.2%)	12 (63.2%)	13 (38.2%)	
Melanoma-molGPA (*n* = 100)				
0–1	23 (23.0%)	9 (22.5%)	14 (23.3%)	0.15
1.5–2	51 (51.0%)	25 (62.5%)	26 (43.3%)	
2.5–3	24 (24.0%)	6 (15.0%)	18 (30.0%)	
3.5–4	2 (2.0%)	0	2 (3.3%)	
Missing	6	2	4	

BM: brain metastases, LDH: lactate dehydrogenase, ECOG PS: Eastern-Cooperative-Oncology-Group Performance Status, Melanoma-molGPA = updated Graded Prognostic Assessment index. Bold police: *p* value statistically significant, < 0.05. *: Inaugural BM means presence of BM at the time of the melanoma’s diagnosis. **: Metachronous BM means absence of BM at the time of the melanoma’s diagnosis, differed apparition of BM.

**Table 4:** Systemic treatment received at least one time after diagnosis of brain metastasis according to the local treatment.

	No LT. (*n* = 42)	SRT (*n* = 28)	WBRT (*n* = 21)	Surgery (+/−RT) (*n* = 16)
No systemic treatment	12 (28.6%)	5 (17.9%)	4 (19.0%)	2 (12.5%)
At least one line of ICI	21 (50.0%)	15 (53.6%)	12 (57.1%)	13 (61.9%)
At least one line of anti-BRAF and/or anti-Mek	12 (28.6%)	10 (35.7%)	9 (42.9%)	7 (33.3%)
At least one line of chemotherapy	5 (11.9%)	7 (25.0%)	5 (23.8%)	3 (14.2%)
Only chemotherapy	2 (4.8%)	2 (7.1%)	2 (9.5%)	0 (0.0%)

LT: local treatment, WBRT: whole brain radiation therapy, SRT: stereotatctic radiosurgery, RT: radiation therapy (i.e., SRT or WBRT), ICI: immune checkpoint inhibitor.

**Table 5:** Radiation therapy related side-effects, grades and corresponding treatment.

	Total Radiation Therapy Patients *n* = 58
Late toxicities	
Radionecrosis	
All grade	6
Grade 1	6
Grade 2	2
Grade 3	3
Treatment for radionecrosis	
Steroids	4
Surgery	1
Radio-induced Oedema	
All grade	9
Grade 1	0
Grade 2	4
Grade 3	5
Treatment for radio-induced Oedema	
Steroids	9
Anticonvulsant	9
Haemorrhagic transformation	
All grades	0

## Data Availability

Data available on request due to restrictions e.g., privacy or ethical. The data presented in this study are available on request from the corresponding author. The data are not publicly available due to restrictions e.g., privacy.
